# Supplementary figures and images for: Dynamic Shifts in Antibiotic Residues and Gut Microbiome Following Tilmicosin Administration to Silkie Chickens
Source: Animals (Basel). 2024 Nov 27;14(23):3428. doi: 10.3390/ani14233428 (PMC11640304; doi:10.3390/ani14233428)

Figure S1

A

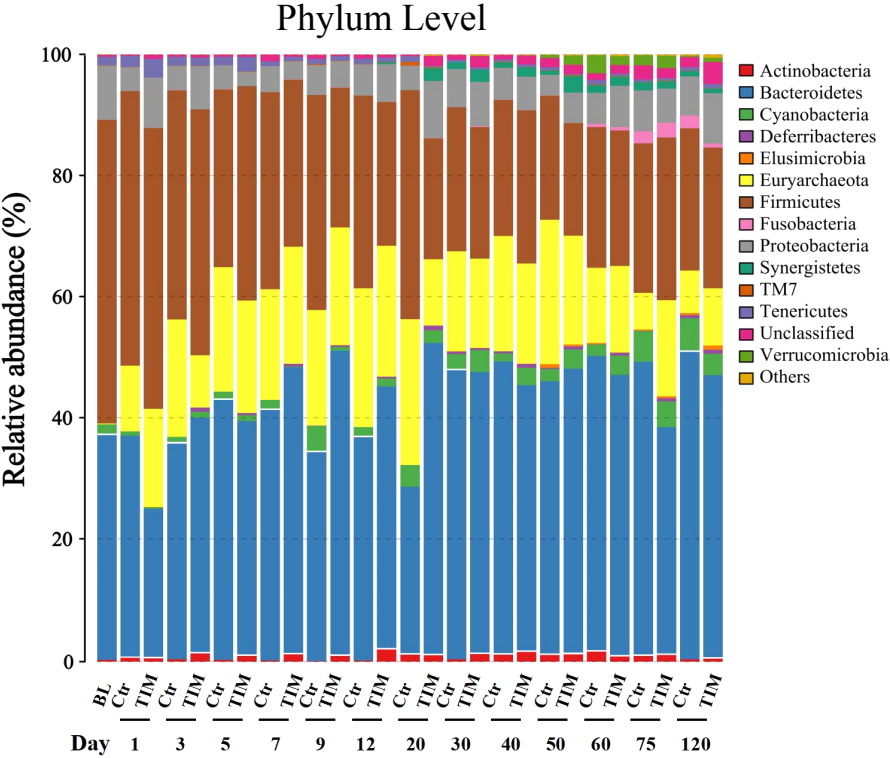

B

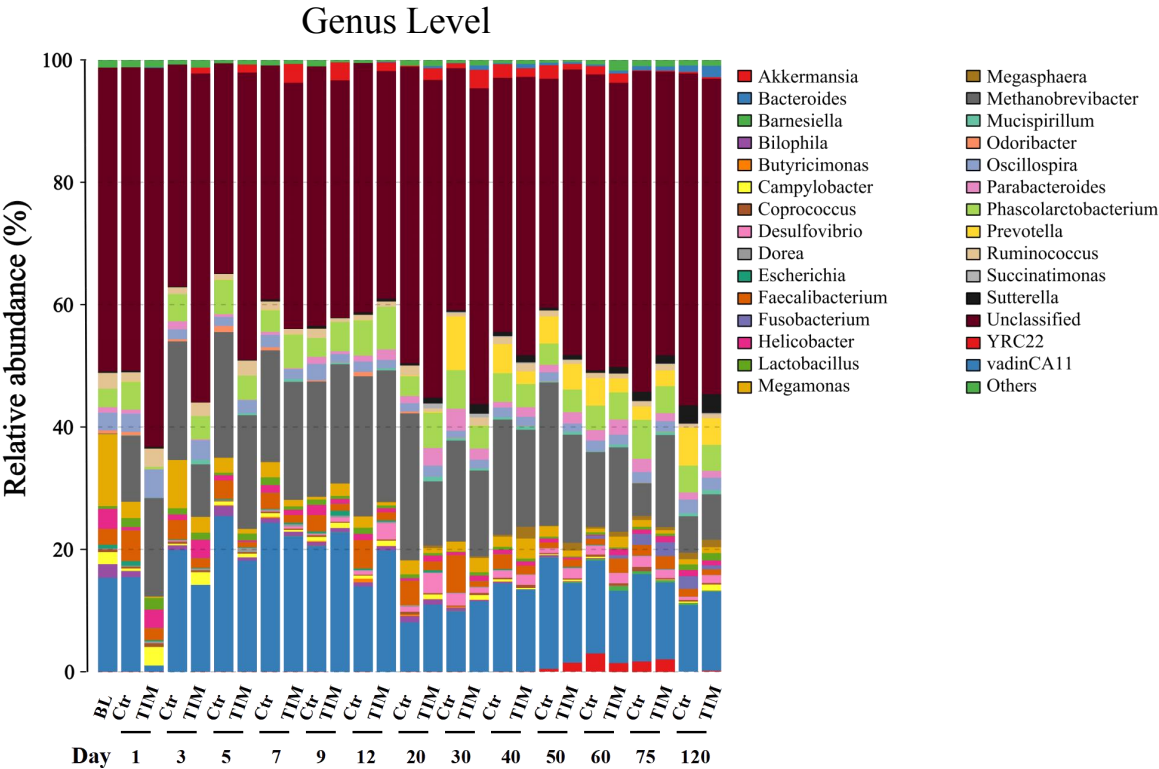

Supplement: Supplementary file 1 [file animals-14-03428-s001.zip › animals-3251781-supplementary.pdf]
